# Comprehensive Assessment of Silver Bioaccumulation and DNA Damage Effects in *Coturnix coturnix japonica* via Blood, Feather, and Egg Using Two Different Sources

**DOI:** 10.3390/ani15233370

**Published:** 2025-11-21

**Authors:** Hanan Al-Khalaifah, Nudrat Fatima, Shabana Naz, Babar Maqbool, Rifat Ullah Khan, Ankqash Ayyub, Muhammad Usama, Swaira Ashfaq, Hifza Shehzadi, Sania Satti, Ala Abudabos, Ibrahim A. Alhidary

**Affiliations:** 1 Environment and Life Sciences Research Center, Kuwait Institute for Scientific Research, Safat 13109, Kuwait; 2 Department of Zoology, Government College University, Faisalabad 38000, Pakistan; 3 Department of Veterinary Medicine, Faculty of Veterinary and Animal Science, University of Agriculture, Dera Ismail Khan 38040, Pakistan; babar.maqbool@uad.edu.pk; 4 Physiology Lab, College of Veterinary Sciences, Faculty of Animal Husbandry and Veterinary Sciences, The University of Agriculture, Peshawar 25100, Pakistan; rukhan@aup.edu.pk; 5 Department of Food and Animal Sciences, College of Agriculture, Tennessee State University, Nashville, TN 37209, USA; 6 Department of Animal Production, College of Food and Agriculture Sciences, King Saud University, Riadh 11451, Saudi Arabia

**Keywords:** Japanese quail, silver nanoparticles, silver nitrate, genotoxicity, comet assay

## Abstract

This study examined the effects of Ag-NPs and AgNO_3_ on silver accumulation in the blood, feathers, eggshells, and egg contents of Japanese quails, as well as their potential to cause DNA damage. A total of 480 (fourteen-day-old) quails were divided into five groups of 96 birds each, arranged into six replicates of sixteen birds with a sex ratio of four males to twelve females. Birds were housed in cages measuring 120 × 60 × 50 cm, and the trial lasted for 65 days. At the end of the trial, six birds per replicate (36 birds per group) were randomly selected and slaughtered for sample collection. Blood was collected via wing vein puncture, feathers were plucked from the breast region, and eggs were collected daily for analysis of eggshell and internal contents. The first group served as a control and was fed a basal diet, while the second and third groups received Ag-NPs at doses of 10 mg/kg and 20 mg/kg, respectively. The fourth and fifth groups were given AgNO_3_ at the same concentrations. The results showed that the highest silver accumulation occurred in all tissues in quails fed the higher dose of Ag-NPs. The greatest accumulation was observed in the eggshells, likely due to their porous structure, which facilitates metal deposition. Both Ag-NPs (20 mg/kg) and AgNO_3_ (10 and 20 mg/kg) induced DNA damage, although the damage was more severe in the groups exposed to Ag-NO_3_. A positive correlation was observed between treatment groups and comet assay parameters, indicating increased DNA fragmentation in exposed birds. In conclusion, the study demonstrated that although Ag-NPs resulted in higher silver accumulation, they caused less DNA damage compared to silver nitrate. These findings highlight that nanoparticulate silver may present a less genotoxic alternative to ionic silver forms for use in poultry systems. Future studies should focus on long-term exposure effects, molecular pathways of oxidative stress and DNA repair, and the safe threshold levels of Ag-NPs to optimize their use in animal nutrition.

## Simple Summary

Silver is widely used in animal production due to its antimicrobial properties, but its accumulation and safety in poultry remain concerns. This study compared silver nanoparticles (Ag-NPs) and silver nitrate (AgNO_3_) for their effects on silver accumulation and DNA damage in Japanese quails. Birds fed higher levels of Ag-NPs showed greater silver deposition, especially in eggshells, but less DNA damage compared with those fed AgNO_3_. These findings suggest that while Ag-NPs accumulate more in tissues, they may be a safer alternative to ionic silver sources, reducing the potential genotoxic effects in poultry.

## 1. Introduction

Nanotechnology involves the manipulation of materials at the nanometer scale, typically between 1 and 100 nanometers. This innovative field has had a significant impact across various industries, including healthcare, agriculture, and the food and nutrition sectors [1,2,3,4]. Researchers are increasingly exploring the unique properties of nanostructures, such as enhanced electrical, magnetic, and optical characteristics, which have considerable implications for improving the nutritional value and bioavailability of food ingredients [5,6,7,8]. A fundamental component of nanotechnology is the use of nanoparticles—extremely small particles with a high surface-area-to-volume ratio [9,10,11]. This feature allows nanoparticles to interact efficiently with biological systems, resulting in improved absorption, reactivity, and targeted delivery of nutrients. Among various nanomaterials [2,5,6,12,13,14], silver nanoparticles (Ag-NPs) are particularly notable due to their remarkable antibacterial, antiviral, and antifungal properties, which contribute to food preservation and safety enhancement [15].

Silver (Ag) exists in both organic and inorganic forms, each with distinct properties and biological behaviors. In its inorganic form, it is commonly used as elemental silver or silver salts, while in its organic form, it forms complexes with organic molecules [10,16]. Ag-NPs are widely used in biomedicine, including in antimicrobial treatments, cancer therapy, diabetes management, wound healing, and biosensing applications due to their high reactivity and surface plasmon resonance properties. However, increasing use of Ag-NPs in consumer and agricultural products raises serious concerns about their environmental persistence and biological safety.

A recent advancement in nanoparticle synthesis is the adoption of green synthesis methods using biological agents such as plant extracts and microorganisms. These methods are safer, cost-effective, and environmentally friendly alternatives to conventional chemical synthesis [17]. One plant of particular importance in green synthesis is *Azadirachta indica* (neem), known for its potent medicinal properties, including antibacterial, antiviral, antifungal, and anti-inflammatory effects [18,19,20]. Utilizing neem extract in nanoparticle synthesis enhances biocompatibility and reduces chemical toxicity, providing a sustainable route for producing functional nanoparticles. Despite these benefits, limited information exists regarding the biological fate and potential toxicity of green-synthesized Ag-NPs in living organisms, especially in avian species.

Ag-NPs have been reported to bioaccumulate in various tissues, potentially exerting toxic effects through oxidative stress and genotoxicity mechanisms [19,21]. Their small size and physicochemical reactivity allow easy penetration through biological membranes, leading to accumulation in organs such as the liver, kidney, and reproductive tissues [22]. Unlike ionic silver (Ag^+^) from compounds such as silver nitrate (AgNO_3_), Ag-NPs can persist in biological systems for longer durations and induce DNA damage and cellular oxidative stress. These effects are of particular concern in birds like Coturnix coturnix japonica (Japanese quail), which serve as suitable model organisms for studying environmental toxicity due to their sensitivity, short lifecycle, and relevance to food chains [23].

Despite growing interest in Ag-NPs, few studies have compared the bioaccumulation and genotoxic potential of chemically synthesized versus green-synthesized Ag-NPs in avian models. The current knowledge gap regarding their relative safety and tissue distribution limits our understanding of their potential ecological and health risks. Therefore, the present study was designed to comparatively assess the bioaccumulation of silver and the associated DNA damage in blood, feathers, and eggs of Japanese quails exposed to two different silver sources (green-synthesized Ag-NPs and AgNO_3_). It was hypothesized that green-synthesized Ag-NPs would exhibit reduced bioaccumulation and lower genotoxic potential compared with the inorganic silver source due to their enhanced biocompatibility and natural capping by phytochemicals.

## 2. Materials and Methods

### 2.1. Synthesis of Silver Nanoparticles

Fresh neem (*Azadirachta indica*) leaves were obtained from mature trees in the Botanical Garden of GCUF, Faisalabad, Pakistan. The leaves were first thoroughly washed with tap water, followed by distilled water, and then completely dried. Once dried, the leaves were cut into small pieces and ground into a fine paste using a mortar and pestle. A 10 g sample of the paste was then heated in 100 mL of distilled water at 60 °C for 30 min to extract the bioactive compounds. After cooling, the extract was filtered through Whatman No. 1 filter paper to remove any solid residues [24]. A 1 mM stock solution of silver nitrate (AgNO_3_; purity ≥ 99%, Sigma-Aldrich, St. Louis, MO, USA) was prepared by dissolving the appropriate amount in distilled water. Subsequently, 10 mL of the neem leaf extract was added to 200 mL of the AgNO_3_ solution and gently stirred on a hot plate. The mixture was maintained at 60 °C for 30 min. A visible color change from yellow to dark red confirmed the successful synthesis of silver nanoparticles (Ag-NPs), indicating the reduction of silver ions and nanoparticle formation [25].

The synthesized Ag-NPs were characterized using a UV-Visible spectrophotometer (Hitachi U-2800, Minato-ku, Toranomon, Japan), with absorbance measured in the 300–600 nm range to monitor nanoparticle formation [19]. Fourier Transform Infrared (FTIR) spectroscopy (PerkinElmer, Waltham, MA, USA) was used to identify the functional groups involved in stabilizing the Ag-NPs. Measurements were taken across a spectral range of 400 to 4000 cm^−1^ with a resolution of 4 cm^−1^. The FTIR analysis showed absorption bands corresponding to carbonyl groups from proteins, indicating that proteins in the neem extract likely capped and stabilized the Ag-NPs. X-ray diffraction (XRD) analysis was performed using an X’Pert Pro diffractometer with Cu Kα radiation to determine the amorphous structure and size distribution of the nanoparticles. Scanning was conducted at a rate of 10°/min. The XRD patterns confirmed an amorphous structure [26]. To ensure colloidal stability during the experimental period, measurements of hydrodynamic diameter (via dynamic light scattering, DLS), polydispersity index (PDI), and zeta potential were carried out at the time of synthesis and at regular 15-day intervals up to the end of the trial. These monitoring steps help confirm that nanoparticles remain stable (i.e., minimal aggregation or surface charge drift) and maintain dose equivalence throughout the experiment [27,28].

### 2.2. Trial Birds and Research Methodology

This study was conducted in accordance with the ethical guidelines for the handling and care of laboratory animals established by Government College University Faisalabad (GCUF). A total of 480 (14-day-old) Japanese quails were acquired and acclimatized for 14 days under controlled conditions, which included a temperature range of 20–25 °C, 70% humidity, and a 16 h light/8 h dark cycle. The quails were housed in wire cages with free access to a commercial basal diet [Table 1] and fresh water. The birds were randomly assigned to five experimental groups, each consisting of 96 birds and six replicates (16 birds per replicate). The groups were as follows: the first group (control) received only the basal diet; the second and third groups received Ag-NPs at 10 mg/kg and 20 mg/kg body weight, respectively; and the fourth and fifth groups received AgNO_3_ at 10 mg/kg and 20 mg/kg body weight, respectively. Treatments were administered weekly via oral gavage over a 65-day period throughout the trial.

The chosen Ag-NP doses (10 and 20 mg/kg) were selected based on previously published poultry and avian studies that reported biological effects and safety at similar levels (e.g., 10 and 20 mg/kg via drinking water or feed) and thereby provide a relevant comparative framework for the present work [29,30]. Lower doses have been reported to elicit minimal physiological responses, whereas substantially higher doses can cause tissue accumulation and adverse effects in some models; therefore, the present levels were intended to span an efficacious yet cautious range.

Treatments were administered by oral gavage once weekly for 65 days to ensure precise and uniform dosing to each bird while minimizing handling frequency and related stress compared with daily gavage. Weekly administration is consistent with other chronic nanoparticle exposure and transfer studies designed to model repeated exposure and to avoid overdosing from continuous high-frequency administration [31,32]. To ensure exposure validity and dose equivalence, particle suspensions were freshly prepared prior to each administration, and nanoparticle stability (hydrodynamic diameter, polydispersity index, and zeta potential) was monitored at synthesis and at regular 15-day intervals throughout the experiment using dynamic light scattering (DLS) and electrophoretic light scattering. These stability checks were incorporated because Ag-NPs can undergo aging, aggregation, and surface charge changes during storage, which affect bioavailability and biological activity [28,33]. Any significant changes in size, PDI, or zeta potential would have prompted re-standardization of the dosing suspension and were recorded in Section 2.

### 2.3. Blood and Feather Sample Collection

At the conclusion of the trial (day 65), 2–3 mL blood samples were collected from the brachial vein of twelve birds per replicate. Six samples were placed in lavender-topped tubes for comet assay analysis, and Six in gold-topped tubes for silver bioaccumulation measurement, following the method described by Khalid et al. [34]. All blood samples were stored at −20 °C until chemical analysis. The birds were then euthanized, and chest feathers were collected post-dissection. These feathers were selected as representative samples, as they are considered to best reflect environmental exposure.

### 2.4. Collection of Eggs

Eggs were collected daily during the quail breeding season, coinciding with the typical morning laying period. To minimize damage, at least 20 eggs per replicate were collected manually. Each egg was labeled with the date and replicate number, then stored in chemically cleaned jars at 4 °C to prevent contamination. Prior to processing, eggs were washed with demineralized water, if required. The eggshells were carefully removed, and the contents were transferred to sterile containers via clean glass dishes. These containers were then frozen, preparing the samples for subsequent chemical analysis [35].

### 2.5. Silver Bioaccumulation and Screening Sample Digestion

Blood samples, collected in gold-topped tubes, were allowed to clot. Serum was then separated from red blood cells (RBCs) by centrifuging the coagulated blood at 2000 rpm for 10 min, maximizing serum yield. A 1:10 serum dilution was prepared by mixing 1 mL of serum with 10 mL of demineralized water [23]. Feather samples were cleaned with acetone, followed by three washes with water. After drying in an oven at 60 °C for two days, the feathers were fragmented. A 1 g portion of each sample was then digested using a 5 mL hydrogen peroxide (H_2_O_2_) and 5 mL nitric acid (HNO_3_) mixture on a 70 °C hot plate. Once digestion was complete, the mixture was cooled to room temperature and filtered through Whatman filter paper No. 1. The filtered solution was then diluted with 25 mL of deionized water [35].

Eggs were cleaned with acetone and demineralized water to remove surface contaminants. Egg contents were extracted using a toothpick and placed in Petri dishes, while eggshells were placed in separate dishes. Samples were dried in an oven to achieve uniform dry matter. Dried eggshells and egg contents were then ground into homogeneous powders. A 0.5 g portion of each powder was mixed with 10 mL of nitric acid (HNO_3_) in conical flasks and heated to 140 °C until the solution became clear [34]. After cooling, the digests were filtered through Whatman filter paper No. 1 and diluted to 25 mL with deionized water in polypropylene flasks. Digested samples were stored at 4 °C until silver content analysis via Atomic Absorption Spectrophotometry [36]. Calibration standards were prepared using certified silver standard solutions (AgNO_3_) to construct calibration curves (R^2^ > 0.999). The detection limit for silver was 0.01 mg/L, and quality control was maintained through analysis of reagent blanks, duplicate samples, and spiked recovery tests, which consistently yielded recovery rates between 95 and 103%. Instrument calibration was verified after every 10 samples to ensure analytical accuracy and reproducibility.

### 2.6. Chemical Analysis of Samples

Silver bioaccumulation in blood, feather, and egg samples was determined using Atomic Absorption Spectrometry (Aurora AI 1200, Vancouver, BC, Canada). Silver concentrations were analyzed at a wavelength of 328.1 nm.

### 2.7. Calculation of Silver Concentration

Silver concentrations were determined using the formula: Metal concentration = (Spectrophotometric reading × Dilution factor)/Sample weight. A dilution factor of 25 mL/g was applied to solid samples (feathers, eggshells, and egg contents), while a factor of 10 mL/mL was used for serum samples.

### 2.8. DNA Damage Examination

**Figure 1:**
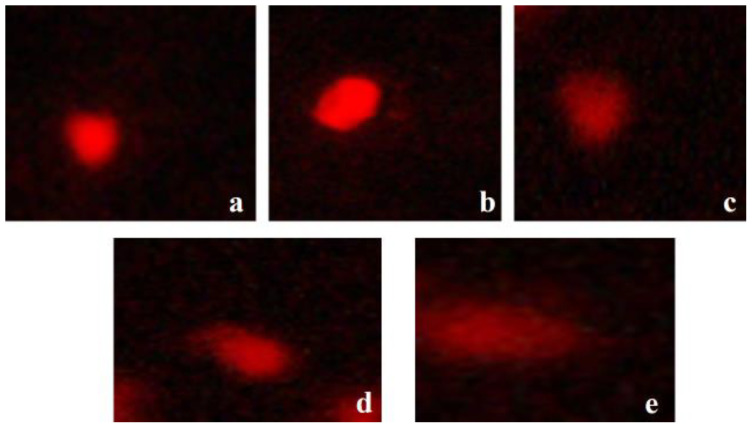
Microscopic images of *Coturnix japonica*) blood samples after comet assay (**a**) subjected to basal diet (control group) (**b**) processed with Ag-NPs (10 mg/kg) (**c**) processed with Ag-NPs (20 mg/kg) (**d**) exposed to AgNO_3_ (10 mg/kg) (**e**) exposed to AgNO_3_ (20 mg/kg).

To assess DNA damage resulting from silver exposure, the Comet assay, also known as Single-Cell Gel Electrophoresis (SCGE), was conducted. The procedure adhered to the methodology outlined by Singh et al. [37] (Figure 1). Image analysis and data acquisition for seven comet parameters—head length, tail length, total comet length, percentage of DNA in the tail and head, tail moment, and olive tail moment—were performed using Casp_1.2.3b1 software. To ensure consistency, microscope settings (exposure, magnification, and contrast) were standardized across all samples, and image acquisition was performed using a high-resolution CCD camera coupled with a fluorescence microscope. Furthermore, the analysis was based on 50 randomly selected nucleoids per slide, avoiding overlapping or damaged cells, and all evaluations were performed by a single trained observer blinded to treatment identity to minimize subjective bias. These procedures ensured the reproducibility and reliability of DNA damage quantification across all experimental groups.

### 2.9. Statistical Analysis

Data analysis was carried out using IBM SPSS Statistics 25 and Statistics 8.1. One-way ANOVA was used to assess silver levels in blood, feathers, eggshells, and egg contents, as well as to evaluate DNA damage in quails exposed to varying concentrations of Ag-NPs and AgNO_3_. To determine which groups differed significantly, Tukey’s post hoc test was used. Furthermore, Pearson’s correlation analysis was performed to examine relationships between comet assay parameters and the different treatment groups of *C. japonica* receiving low and high doses of Ag-NPs and AgNO_3_. Before conducting ANOVA, data were tested for normality using the Shapiro–Wilk test and for homogeneity of variances using Levene’s test. Only data that met these assumptions were subjected to parametric analysis; otherwise, appropriate data transformations were applied. The statistical model included treatment as a fixed effect, while experimental replicates were considered random effects to account for potential variation among replicates. All data were expressed as mean ± standard deviation (SD), and significance was declared at *p* < 0.05.

## 3. Results

**Figure 2:**
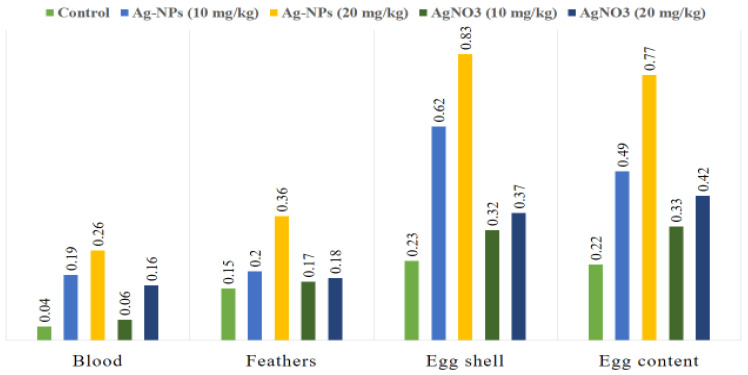
Concentration of Silver (μg/g) in the blood, feather and egg shell, and egg content samples of *Coturnix japonica* supplemented with low and high doses of Ag-NPs and AgNO_3_(Ag-NPs = Silver nanoparticles; AgNO_3_ = Silver Nitrate).

Table 2 shows that silver concentrations in *Coturnix japonica*) differed significantly depending on the type and dosage of silver administered (*p* < 0.05). The highest accumulation was observed in blood, feathers, eggshells, and egg contents of quails fed a high dose of Ag-NPs, whereas moderate increases occurred with a lower dose. Birds treated with AgNO_3_ showed intermediate levels, and the control group consistently had the lowest accumulation. Superscript letters denote statistically significant differences among groups (*p* < 0.05). Overall, silver accumulation followed the order: eggshells > egg contents > feathers > blood, with the highest levels in the high-dose Ag-NPs group (Figure 2). The treatment ranking was: Ag-NPs (high dose) > Ag-NPs (low dose) > AgNO_3_ (high dose) > AgNO_3_ (low dose) > control (*p* < 0.05).

All comet assay parameters (Lhead, Ltail, Lcomet, HeadDNA, TailDNA, TM, OTM) varied significantly among treatments (*p* < 0.05; Table 3). The AgNO_3_ high-dose group exhibited the greatest DNA damage, while Ag-NPs (low and high dose) produced comparatively lower values, indicating reduced genotoxicity. Microscopic observations confirmed significant differences (*p* < 0.05), as illustrated in Figure 1.

**Figure 3:**
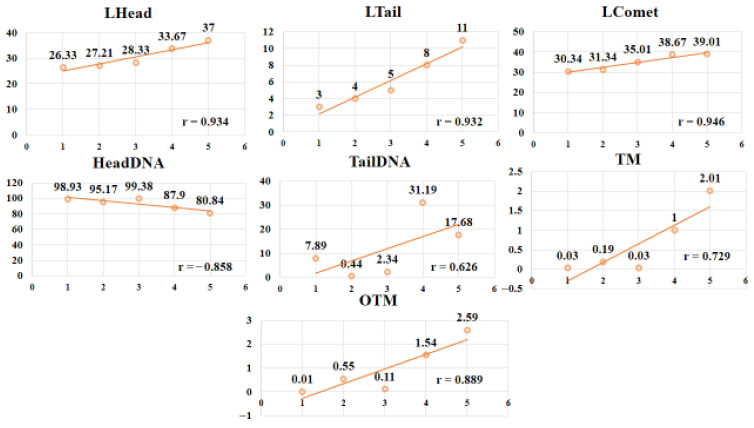
Correlation analysis between comet assay parameters and different treatment groups of *Coturnix japonica* exposed to low and high levels of Ag-NPs and AgNO_3_. (Ag-NPs = Silver nanoparticles; AgNO_3_ = Silver nitrate; 1 = Control; 2 = Ag-NPs (10 mg/kg); 3 = Ag-NPs (20 mg/kg); 4 = AgNO_3_ (10 mg/kg); 5 = AgNO_3_ (20 mg/kg).

Correlation analysis revealed strong positive associations between silver exposure and comet parameters, including Lhead (r = 0.934, *p* < 0.01), Ltail (r = 0.932, *p* < 0.01), Lcomet (r = 0.946, *p* < 0.01), TailDNA (r = 0.626, *p* < 0.05), TM (r = 0.729, *p* < 0.05), and OTM (r = 0.889, *p* < 0.01). Conversely, HeadDNA showed a strong negative correlation (r = –0.858, *p* < 0.01) with treatment groups (Figure 3). These findings suggest that silver compounds, particularly AgNO_3_, significantly compromised DNA integrity, with damage severity increasing at higher concentrations.

## 4. Discussion

In the present study, the results showed that the mean values of Ag varied significantly in quails treated with different concentrations of Ag-NPs and AgNO_3_. Overall, the key findings indicate that high-dose Ag-NPs led to the greatest accumulation in all tissues, particularly eggshells, whereas AgNO_3_ induced moderate accumulation, and controls had the lowest levels. DNA damage was more severe with AgNO_3_ exposure, while Ag-NPs, even at higher doses, produced comparatively lower genotoxic effects. Correlation analyses confirmed strong associations between tissue silver levels and comet assay parameters, supporting a dose- and form-dependent impact on DNA integrity. These findings highlight both the bioaccumulation potential of silver nanoparticles and their relatively lower genotoxicity compared to ionic silver. Because blood reflects overall health and plays a crucial role in transporting nutrients, metabolic waste, and hormones, this elevated level is especially significant [38]. It indicates enhanced absorption and retention of silver in nanoparticle form, likely due to the small size and unique physicochemical properties of Ag-NPs, which promote their passage across biological membranes. Indeed, Ag-NP coatings can modulate ion release and membrane interaction, increasing bioavailability compared to ionic forms [39]. The enhanced bioaccumulation of Ag from Ag-NPs may be attributed to their nanoscale dimensions and high surface area-to-volume ratio, which increase their reactivity and interaction with biological tissues. Ag-NPs can penetrate epithelial barriers through endocytosis or diffusion, allowing them to enter systemic circulation more efficiently than ionic silver (Ag^+^) from AgNO_3_ [40]. Once in the bloodstream, Ag-NPs may bind to plasma proteins or cellular membranes, leading to prolonged circulation time and accumulation in various tissues. Additionally, the slow dissolution of Ag-NPs into Ag^+^ within biological environments may contribute to sustained silver release, thereby enhancing systemic retention and potential biological effects [41]. Moreover, nanoparticle aggregation and ligand dissociation dynamics may influence Ag-NP stability and ion release in biological matrices, as previously demonstrated by Luo et al. [42], where competitive binding of capping ligands affected nanoparticle aggregation and metal bioavailability.

However, it is important to note that inter-individual variation in absorption efficiency, metabolic rate, and hepatic detoxification capacity among quails could have influenced the observed accumulation patterns. Moreover, potential dietary interactions—such as the binding of silver ions with feed minerals, amino acids, or antioxidants—may have modulated the bioavailability and systemic distribution of silver, acting as confounding factors in the interpretation of tissue Ag levels.

Significant differences were observed in the mean Ag concentrations in the feathers of Japanese quails administered varying doses of Ag-NPs and AgNO_3_. The highest Ag level was detected in the feathers of birds receiving a high dose of Ag-NPs. Once in systemic circulation, Ag-NPs are more likely to persist in the bloodstream and reach peripheral tissues compared to ionic silver from AgNO_3_. As feathers develop, they incorporate circulating trace elements, such as silver, into their keratin matrix. The prolonged presence of Ag-NPs in the body increases the likelihood of their deposition in feather tissue during growth, resulting in higher silver concentrations [43]. Feathers were selected as a marker for bioaccumulation due to their ability to reflect internal metal exposure over extended periods. Feathers serve as excretory pathways for metals, and their accumulation indicates chronic exposure over time, as opposed to blood, which reflects more recent exposure events [44,45]. This is consistent with findings from Khalid et al. [34], who reported selenium accumulation patterns in quail tissues, reinforcing feathers and eggs as reliable bioindicators. Moreover, feathers can be collected non-invasively, enabling repeated sampling without harming the animal. During feather formation, trace elements present in the bloodstream become permanently embedded in the keratin structure, providing a stable and time-integrated record of exposure. The strong binding affinity of metals to keratin further preserves the elemental composition, making feathers an effective tool for long-term monitoring of environmental contaminants in avian species [46]. Nevertheless, factors such as oxidative stress status, molting cycle, or individual variation in keratin synthesis could have partially influenced metal incorporation into feather tissues, introducing biological variability that may obscure dose-dependent relationships.

Our results indicated that increasing dietary concentrations of Ag-NPs and AgNO_3_ from 10 to 20 mg/kg feed led to a significant accumulation of silver in both the eggshell and internal contents of Japanese quail eggs. This suggests that silver, particularly in nanoparticle form, can cross biological barriers and be transferred from the hen to the developing egg. Eggshells serve as both a protective barrier and a mineral reservoir, often accumulating metals through maternal deposition during shell formation [36]. The incorporation of silver into the eggshell may alter its structural integrity, while the presence of silver in the egg contents indicates potential for vertical transmission and raises concerns about embryotoxic effects [47]. Similar patterns of metal accumulation in eggshells and egg contents have been reported in other avian species, indicating that nanoparticle and ionic metals can be vertically transmitted across diverse bird species [34,48,49]. Furthermore, oxidative stress and antioxidant status play a critical role in mediating the impact of metal accumulation on egg quality. Dietary antioxidants have been shown to maintain egg quality and reduce oxidative damage, suggesting that maternal antioxidant defense may modulate silver-induced effects in eggs [50]. It should also be considered that nutrient composition and mineral balance in the diet could have modulated metal transport through the oviduct and shell gland, influencing the extent of Ag deposition in eggshells and contents.

The greatest concentration of Ag was detected in the eggshells of quails, compared to the levels found in egg contents, feathers, and blood samples. The eggshell is primarily composed of calcium carbonate crystals, along with a matrix of proteins and other minerals, which are deposited by specialized cells in the shell gland during egg formation [51]. This mineralization process allows the eggshell to serve as a reservoir for various minerals and metals circulating in the hen’s bloodstream, including silver. Metals like silver often have a strong affinity for binding with calcium and other mineral components due to similar chemical properties, facilitating their incorporation into the eggshell’s crystalline matrix. This selective deposition means that metals can accumulate in the shell in higher amounts than in other tissues [52]. Additionally, the eggshell functions as a protective barrier to shield the developing embryo from harmful substances, including toxic metals. By sequestering silver in the shell, the bird’s physiology limits the amount that reaches the internal contents of the egg, such as the yolk and albumen, thereby reducing potential toxicity to the embryo [53]. However, physiological factors such as laying sequence, calcium metabolism, and oxidative balance may have further contributed to inter-sample variation, acting as potential confounders in metal deposition outcomes.

Recent pathological studies examining the genotoxic effects of Ag-NPs and AgNO_3_, confirmed by comet assays, revealed that DNA damage was significantly more pronounced in samples exposed to AgNO_3_ compared to those treated with Ag-NPs or control groups. This finding highlights the critical role of silver’s chemical form in its genotoxic potential. AgNO_3_ is a soluble salt that rapidly dissolves in biological fluids, releasing silver ions (Ag^+^) almost immediately. These free silver ions are highly reactive and can readily penetrate cells, where they interact directly with vital biomolecules such as DNA [41]. In contrast, biofunctionalized nanoparticles, including surface-modified or ligand-coated systems, can influence cellular uptake, reduce direct ion release, and target specific tissues, thereby modulating bioaccumulation and genotoxic effects. For instance, aptamer-AuNP-conjugated carboxymethyl chitosan-functionalized graphene oxide systems have demonstrated controlled interaction with biological targets, highlighting how functionalization strategies can improve nanoparticle safety and specificity in biological matrices [54]. Inside the cell, silver ions promote the formation of reactive oxygen species (ROS), including free radicals and peroxides. These ROS attack the DNA molecule, causing oxidative damage such as single- and double-strand breaks, base modifications, and crosslinking. The accumulation of such damage compromises the integrity of genetic material and may lead to mutations or cell death. At the molecular level, Ag^+^-induced oxidative stress may also influence DNA methylation and impair repair pathways, as reported by Yang et al. [55]. In contrast, Ag-NPs are solid particles that release silver ions more slowly due to their particulate nature and surface chemistry [56]. This gradual ion release results in fewer free silver ions being immediately available to interact with DNA, thereby reducing the extent of direct damage. Furthermore, Ag-NPs are often internalized by cells via endocytosis and become sequestered within cellular compartments like lysosomes, limiting their direct contact with the nucleus, where DNA is located. As a result, while Ag-NPs can still induce oxidative stress and cellular damage, the severity and immediacy of DNA damage tend to be lower than those caused by silver ions from AgNO_3_ [57]. This difference in genotoxicity is consistent with the potent nature of silver ions, which can directly bind DNA bases, generate ROS, and cause strand breaks and chromosomal aberrations [58,59]. Despite higher silver levels in tissues following Ag-NPs exposure, the DNA damage is comparatively less severe, likely due to the slower ion release and sequestration of nanoparticles within lysosomes or vesicular compartments [60]. Furthermore, endogenous cellular antioxidant systems, including superoxide dismutase (SOD), catalase (CAT), and glutathione peroxidase (GSH-Px), may neutralize reactive oxygen species generated by Ag-NPs, thereby mitigating oxidative damage to DNA and other cellular components. Maternal antioxidant status and dietary antioxidants have also been shown to protect against metal-induced oxidative stress, helping maintain cellular integrity and overall egg quality in avian species [50]. Additionally, cellular antioxidant defenses may be more effective against the oxidative stress induced by nanoparticles, further mitigating DNA damage. Nonetheless, it should be acknowledged that oxidative stress intensity, endogenous antioxidant capacity, and inter-individual genetic variation in DNA repair efficiency may have contributed to variation in comet assay parameters, serving as biological confounders influencing the genotoxic response.

## 5. Conclusions

This study demonstrated that Ag-NPs led to higher silver accumulation in Japanese quails, particularly in eggshells, whereas AgNO_3_ caused more pronounced DNA damage. These results highlight that both the chemical form and dose of silver critically influence bioaccumulation and genotoxicity. While Ag-NPs showed comparatively lower DNA damage under the tested conditions, the findings are limited to short-term exposure in a single species. Future studies should investigate chronic exposure, reproductive and transgenerational effects, and environmental implications to fully assess the safety of silver nanoparticles in poultry and other avian species.

## Figures and Tables

**Table 1:** Composition of Basal Diets Used for Japanese Quails.

Ingredients	Percentage (%)
Soyabean oil	0.50
Soyabean meal	21.60
Wheat	4.10
Calcium phosphate (GR)	1.00
Calcium carbonate (GR)	9.50
Cottonseed meal	2.00
Corn	60.50
Phytases	0.015
Sodium chloride	0.30
DL-methionine	0.08
Vitamin premix	0.15
Mineral premix	0.035
50% Choline chloride	0.10
Experimental additives	0.12
Total	100.0
**Nutrient analysis**	
Methionine + Cysteine (%)	0.55
Apparent metabolizable energy (AME), Mcal/kg	2.57
Se^c^ (mg/kg)	0.056
Crude protein (%)	24.0
Lysine (%)	0.74
Total phosphorus (%)	0.54
Available phosphorus (%)	0.31
Calcium (%)	3.7
Methionine (%)	0.3

Abbreviations: Guaranteed Reagent (GR) is provided per kilogram of diet, including vitamin A at 12,500 International Units (IU), vitamin D3 at 2500 IU, vitamin E at 18.75 milligrams (mg), vitamin K3 at 2.65 mg, vitamin B1 at 2 mg, vitamin B2 at 6 mg, vitamin B12 at 0.025 mg, biotin at 0.325 mg, folic acid at 1.25 mg, and niacin at 50 mg. Additionally, trace minerals provided per kilogram of diet include copper (Cu) at 8 mg, iron (Fe) at 80 mg, zinc (Zn) at 80 mg, manganese (Mn) at 60 mg, and iodine (I) at 1.2 mg.

**Table 2:** Comparative Analysis of Silver Levels (Mean ± SD) in Blood, Feathers, Eggshells, and Egg Contents of *C. japonica* Supplemented with Low and High Doses of Ag-NPs and AgNO_3_.

Samples	Control	Ag-NPs (10 mg/kg)	Ag-NPs (20 mg/kg)	Ag-NO3 (10 mg/kg)	Ag-NO3 (20 mg/kg)	*p* Value
Blood	0.04 ± 0.01 ^d^ (SE = 0.004; 95% CI: 0.03–0.05)	0.19 ± 0.01 ^b^ (SE = 0.004; CI: 0.18–0.20)	0.26 ± 0.01 ^a^ (SE = 0.004; CI: 0.25–0.27)	0.06 ± 0.02 ^d^ (SE = 0.004; CI: 0.25–0.27)	0.16 ± 0.02 ^c^ (SE = 0.008; CI: 0.04–0.08	0.001
Feathers	0.15 ± 0.00 ^d^ (SE = 0.002; CI: 0.15–0.16)	0.20 ± 0.02 ^b^ (SE = 0.008; CI: 0.18–0.22)	0.36 ± 0.01 ^a^ (SE = 0.004; CI: 0.35–0.37)	0.17 ± 0.00 ^cd^ (SE = 0.012; CI: 0.29–0.35)	0.18 ± 0.01 ^c^ (SE = 0.002; CI: 0.16–0.18)	0.001
Egg shell	0.23 ± 0.02 ^e^ (SE = 0.008; CI: 0.21–0.25)	0.62 ± 0.04 ^b^ (SE = 0.016; CI: 0.59–0.65)	0.83 ± 0.02 ^a^ (SE = 0.008; CI: 0.81–0.85)	0.32 ± 0.03 ^d^ (SE = 0.012; CI: 0.29–0.35)	0.37 ± 0.04 ^c^ (SE = 0.012; CI: 0.29–0.35)	0.001
Egg content	0.22 ±0.04 ^e^ (SE = 0.016; CI: 0.18–0.26)	0.49 ± 0.07 ^b^ (SE = 0.028; CI: 0.42–0.56)	0.77 ± 0.04 ^a^ (SE = 0.016; CI: 0.73–0.81)	0.33 ± 0.04 ^d^ (SE = 0.016; CI: 0.29–0.37)	0.42 ± 0.04 ^c^ (SE = 0.016; CI: 0.29–0.37)	0.001

Ag-NPs = Silver Nanoparticles; AgNO_3_ = Silver Nitrate. The mean values with distinct superscripts (^a–e^) in a row exhibit substantial variation at (*p* < 0.05).

**Table 3:** Comet parameters (Mean ± SD) in the blood of C. japonica supplemented with different doses of Ag-NPs and AgNO_3_.

Parameters	Control	Ag-NPs (10 mg/kg)	Ag-NPs (20 mg/kg)	AgNO_3_ (10 mg/kg)	AgNO_3_ (20 mg/kg)	*p* Value
LHead	26.33 ± 2.31 ^c^ (SE = 0.94; CI: 24.3–28.3)	27.21 ± 2.31 ^c^ (SE = 0.94; CI: 25.2–29.2)	28.33 ± 8.33 ^c^ (SE = 3.40; CI: 21.0–35.7)	33.67 ± 4.16 ^b^ (SE = 1.70; CI: 30.1–37.2)	37.00 ± 3.46 ^a^ (SE = 1.41; CI: 33.9–40.1)	0.000
LTail	3.00 ± 0.00 ^b^ (SE = 0.00; CI: 3.0–3.0)	4.00 ± 0.00 ^b^ (SE = 0.00; CI: 4.0–4.0)	5.00 ± 0.00 ^b^ (SE = 0.00; CI: 5.0–5.0)	8.00 ± 1.00 ^a^ (SE = 0.41; CI: 7.1–8.9)	10.67 ± 1.15 ^a^ (SE = 0.47; CI: 9.5–11.8)	0.000
LComet	30.34 ± 2.30 ^c^ (SE = 0.94; CI: 28.3–32.3)	31.34 ± 2.31 ^c^ (SE = 0.94; CI: 29.3–33.3)	35.00 ± 3.61 ^b^ (SE = 1.47; CI: 32.0–38.0)	38.67 ± 4.16 ^a^ (SE = 1.70; CI: 35.1–42.2)	39.00 ± 8.71 ^a^ (SE = 3.56; CI: 31.4–46.6)	0.000
HeadDNA	98.93 ± 0.94 ^a^ (SE = 1.63; CI: 4.3–11.5)	95.17 ± 2.82 ^b^ (SE = 1.15; CI: 92.9–97.4)	99.38 ± 0.26 ^a^ (SE = 0.11; CI: 99.1–99.7)	87.90 ± 7.87 ^c^ (SE = 3.21; CI: 80.9–94.9)	80.84 ± 5.44 ^d^ (SE = 2.22; CI: 75.8–85.8)	0.000
TailDNA	7.89 ± 4.00 ^c^ (SE = 0.01; CI: 0.02–0.04)	0.44 ± 0.41 ^d^ (SE = 0.17; CI: 0.0–0.8)	2.34 ± 2.04 ^d^ (SE = 0.83; CI: 0.7–4.0)	31.19 ± 24.37 ^a^ (SE = 9.95; CI: 11.6–50.8)	17.68 ± 27.42 ^b^ (SE = 11.2; CI: −6.0–41.3)	0.000
TM	0.03 ± 0.02 ^b^ (SE = 0.01; CI: 0.02–0.04)	0.19 ± 0.11 ^b^ (SE = 0.04; CI: 0.09–0.29)	0.03 ± 0.01 ^b^ (SE = 0.00; CI: 0.02–0.04)	1.00 ± 0.67 ^ab^ (SE = 0.27; CI: 0.3–1.7)	2.01 ± 0.45 ^a^ (SE = 0.18; CI: 1.5–2.5)	0.013
OTM	0.01 ± 0.11 ^e^ (SE = 0.05; CI: −0.09–0.11)	0.55 ± 0.32 ^c^ (SE = 0.13; CI: 0.3–0.8)	0.11 ± 0.53 ^d^ (SE = 0.22; CI: −0.4–0.6)	1.54 ± 0.89 ^b^ (SE = 0.36; CI: 0.7–2.4)	2.59 ± 0.48 ^a^ (SE = 0.19; CI: 2.0–3.2)	0.000

Ag-NPs = Silver Nanoparticles; AgNO_3_ = Silver Nitrate; LHead = Length of Head; LTail = Length of Tail; LComet = Length of Comet; TM = Tail Moment; OTM = Olive Tail Moment. The mean values with distinct superscripts (^a–e^) in a row exhibit significant variations at (*p* < 0.05).

## Data Availability

Data is included within the manuscript.
